# A Non-Parametric Peak Calling Algorithm for DamID-Seq

**DOI:** 10.1371/journal.pone.0117415

**Published:** 2015-03-18

**Authors:** Renhua Li, Leonie U. Hempel, Tingbo Jiang

**Affiliations:** 1 State Key Laboratory of Tree Genetics and Breeding, Northeast Forestry University, Harbin 150040, China; 2 Laboratory of Cellular and Developmental Biology, NIDDK/NIH, Bethesda, MD, United States of America; Northwestern University, UNITED STATES

## Abstract

Protein—DNA interactions play a significant role in gene regulation and expression. In order to identify transcription factor binding sites (TFBS) of double sex (DSX)—an important transcription factor in sex determination, we applied the DNA adenine methylation identification (DamID) technology to the fat body tissue of *Drosophila*, followed by deep sequencing (DamID-Seq). One feature of DamID-Seq data is that induced adenine methylation signals are not assured to be symmetrically distributed at TFBS, which renders the existing peak calling algorithms for ChIP-Seq, including SPP and MACS, inappropriate for DamID-Seq data. This challenged us to develop a new algorithm for peak calling. A challenge in peaking calling based on sequence data is estimating the averaged behavior of background signals. We applied a bootstrap resampling method to short sequence reads in the control (Dam only). After data quality check and mapping reads to a reference genome, the peaking calling procedure compromises the following steps: 1) reads resampling; 2) reads scaling (normalization) and computing signal-to-noise fold changes; 3) filtering; 4) Calling peaks based on a statistically significant threshold. This is a non-parametric method for peak calling (NPPC). We also used irreproducible discovery rate (IDR) analysis, as well as ChIP-Seq data to compare the peaks called by the NPPC. We identified approximately 6,000 peaks for DSX, which point to 1,225 genes related to the fat body tissue difference between female and male *Drosophila*. Statistical evidence from IDR analysis indicated that these peaks are reproducible across biological replicates. In addition, these peaks are comparable to those identified by use of ChIP-Seq on S2 cells, in terms of peak number, location, and peaks width.

## Introduction

Systematic identification of transcription factor binding sites (TFBS) contributes to understanding of gene expression and regulation. Due to the complicated protein-DNA interactions, accurate identification of genome-wide TFBS is challenging.

Chromatin immunoprecipitation followed by massively parallel sequencing (ChIP-Seq) has been commonly used in the detection of TFBS [1,2]. This technology relies on a specific antibody to pull down a transcription factor that is cross-linked to the chromatin. To compare signal to background noise, we need a control (input) in which the antibody is not incorporated. Regarding data analysis and peak calling in ChIP-Seq, several steps are often involved; that is, quality check of sequencing data, mapping quality sequence reads to a reference genome, normalization between samples (ChIP vs. input) to adjust for sequencing depth differences, and calling peaks using statistical algorithms, including MACS and SPP [3,4]. These algorithms are based on the idea that reads signals tagging both DNA strands are symmetrically distributed at a binding site [3, 5]. Thus the middle point of the estimated distance between the two distributions of tags is a potential TFBS [5].

An alternative technology is DNA adenine methylation identification followed by deep sequencing (DamID-Seq) [6, 7]. It is based on the idea of fusing a transcription factor to *Escherichia coli* DNA adenine methyltransferase (dam). Expression of this fusion protein *in vivo* results in specific methylation of adenines in the GATC sites surrounding the binding sites [6]. The adenine-methylated DNA fragments are then isolated by a restriction enzyme (*Dpnl*), followed by PCR, library construction, and deep sequencing. To compare the signals of DamID to that expected from the background adenine methylation, we use Dam only as a control in the experiments.

In DamID-seq experiments, there is no guarantee that the induced adenine methylation signals around a TFBS is symmetrical, because the chemical process is affected by many known and unknown factors, for instance, 3-D structure of the chromatin. While the existing algorithms, including SPP and MACS [3, 4] are based on the assumption that there is a symmetrical distribution of signals. Violation of the assumption may result in unstable identification of peaks (will be addressed below). These challenged us to develop a new algorithm.

Double sex (DSX) is a transcription factor that plays a fundamental role in sex determination in *Drosophila melanogaster*. Interesting genes, including yolk protein genes (*yp1* and *yp2*), have been established as direct target genes of DSX [8,9,10]. Recently, the DamID-Seq technology has been used to identify direct targets of the *Drosophila* DSXF protein [10]. In order to identify target genes of both DSXF and DSXM proteins in *Drosophila melanogaster*, we performed DamID-seq against DSX in fat body tissue of 5-day female and male *Drosophila*. In this paper, we focus on the method of peak calling for DamID-Seq data.

## Materials and Methods

### 1. The DamID-Seq experiments

In order to identify DSX biding sites in fat body tissue of 5-day female and male *Drosophila melanogaster*, we used the Dam-DsxF/+, Dam-DsxM /+, and corresponding Dam/+ (control) genotypes to prepare for sequencing libraries. These experiments had two biological replicates per genotype per sex. Dam only, in which only the Dam was used as a surrogate for the fusion protein, was used as a control. Samples of both DamID and Dam only were processed with the same amount of starting DNA. The libraries were sequenced with 76-bp single-end reads on the Illumina GA II platform. Using Bowtie [11], we then aligned quality sequence reads to the *Drosophila* genome sequence (dm3), which was downloaded from the Flybase (http://flybase.org/). The mapping parameters included uniquely mapped reads with up to two mismatches and trimming the primer sequence.

### 2. A new peak calling algorithm for DamID-Seq data

Using computational tools, including FASTQC [12] and Bowtie [11], we performed data quality check, followed by mapping the quality sequence reads onto the *Drosophila* genome. After these, our algorithm comprises the following steps:

#### Step 1: Resampling reads from the Dam only

In DamID-Seq, signals from the Dam only (control) are markedly variable across the *Drosophila* genome (will be shown below), suggesting that expectation of background signals needs to be carefully estimated before peak calling. We hypothesized that the background signals can be estimated by bootstrap resampling through repeated removal of a small fraction of reads (e.g. 10%) from a control sample. To test this hypothesis, we resampled at random 90% of the quality reads by chromosome arms from the control. The resampled reads are then pooled for signal enrichment assessment.

#### Step2: Signal enrichment estimation

Signal enrichment is related to chromosomal regions. As many known transcription factor binding sites are less than 100 bp [4], we binned the *Drosophila* genome into non-overlapping 100-bp running windows. We then used reads per million mapped reads (RPM) to adjustment for coverage differences between a DamID sample and a control (only the resampled sequence reads were considered). After local smoothing using the Gaussian kernel method [13], we finally computed signal enrichment for each window as shown below:

$$y_{i}=\log2(x_{i}/c_{i})$$

where *yi* is the signal enrichment for the *i*th window; *xi* and *Ci* indicate the *i*th window RPM for the Dam and Dam only samples, respectively.

#### Step 3. Filtering signal enrichment

Intuitively, when a signal in the DamID sample is larger than that in the control, there might be a local peak. We selected those windows with positive signal enrichment (or *yi* > 0); that is

$$k=\arg(y_{i}>0)$$

where *k* is a vector of arguments that denotes the positive local signal enrichment measurements.

#### Step 4. Evaluating averaged behavior of the signal enrichment

Among the selected windows, the imputed local signal enrichment varied markedly (will be shown below). Its overall performance is related to the median of the log2 fold changes (MFC) as shown below:

$$MFC=median(y_{i})$$

To estimate distribution of the MFC, we then iterated steps 1 through 4 multiple times (N = 200). We used 95 percentile of the MFC distribution (MFC95) as a cutoff to declare significant windows.

#### Step 5. Calling peaks by merging adjacent significant windows

Distribution of the genome-wide significant windows reveals that they are in clusters, which is expected as the *Dpnl* cutting sites are non-randomly distributed (will be shown below). We then merged the adjacent windows (details will be shown below), in order to call peaks. A flow chart summarizing these steps is given in S1 Fig.

### 3. Irreproducible discovery rate (IDR) analysis

Once we have identified a set of peaks, the next step is to test their consistency between biological replicates. We used the irreproducible discovery rate (IDR) analysis that has been suggested as a standard method [14].

Regarding IDR analysis, we need to lower the threshold, in order to introduce a fraction of false positive peaks [15]. Since intuitively a biological meaningful peak should have a positive *yi* value, we then relax the cutoff to 0. Thus the windows with local signal enrichment corresponding to 0 ≤ *yi* < MFC95 are non-significant. Similar to the significant windows mentioned above, the adjacent non-significant windows are also merged, based on the same criterion that will be shown below. The resultant non-significant peaks are then pooled with the significant peaks for IDR analysis.

We applied IDR analysis to assess peak consistency. Regarding two sets of peaks from the two biological replicates of a genotype, we need to compare corresponding peaks (≥ 50% base pair overlap). The peaks from each of the replicates are then ranked respectively by the peaks’ strength, which is represented by the maximum local signal enrichment of the component windows. We then used the method of Li et al. [15] to find the decay point that marks the start of inconsistency. The peaks before the decay point are claimed to be reproducible [15].

## Results

### 1. Sequencing depth summary

Sequencing depth of each library is summarized in S1 Table. Total number of quality reads is comparable between the samples, especially for Dam-DsxF and the control samples (S1 Table). After aligning the quality reads to the *Drosophila* genome, total mapped reads range from 19 to 34 million across the samples, out of which 12–28 million are uniquely mapped reads (S1 Table). Variation in mapped reads between samples will be adjusted using the RPM as described in the Method section.

### 2. Distributional characteristics of mapped reads

**Fig 1:**
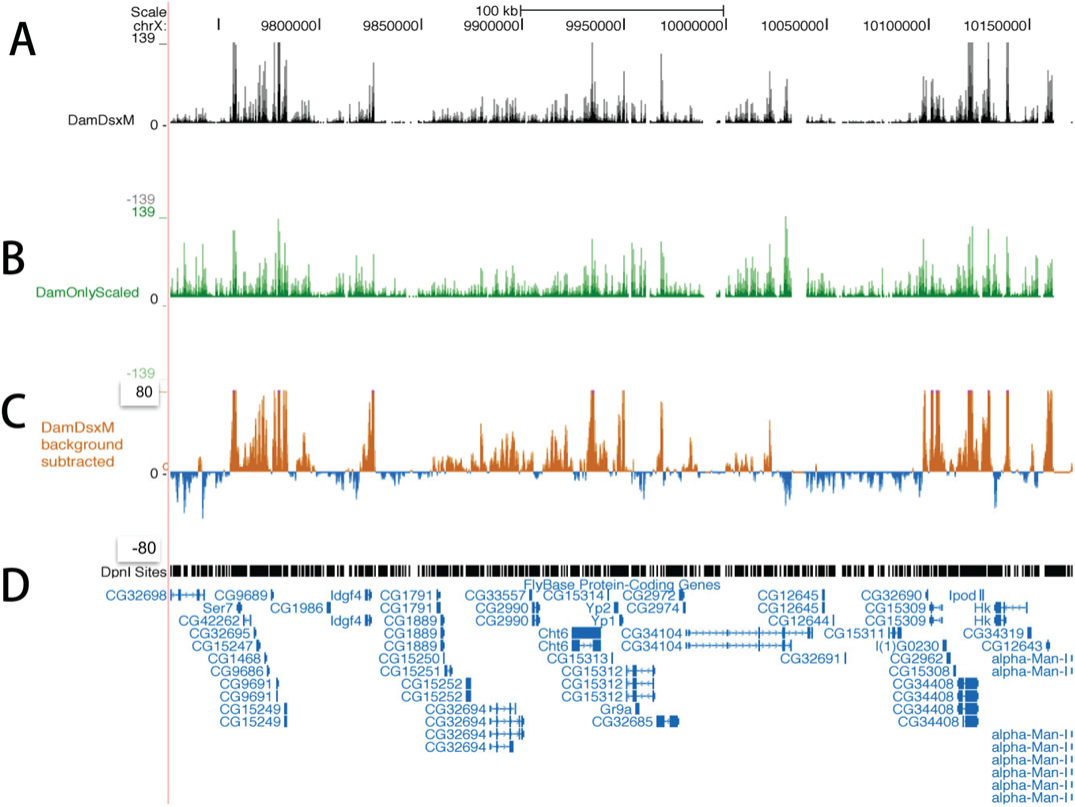
Distribution of uniquely mapped reads on chromosome X of *Drosophila melanogaster*. A snapshot of the University of California, Santa Cruz (UCSC) genome browser shows the distribution of the mapped reads after scaling in reads number per million mapped reads. A and B: The mapped reads in the Dam-DsxM/+ and Dam only (control) genotypes, respectively. C: Reads distribution of Dam-DsxM/+ after the control signal subtraction. D: Distribution of the restriction enzyme *Dpnl*. Below this the annotated genes are displayed.

Distributions of mapped reads in Dam-DsxM and Dam only (control) are illustrated by the *Drosophila* chromosome X (Fig. 1). The reads in the control are non-uniformly distributed and the peaks overlap with that of the DamID (Fig. 1 A&B). After subtraction of background reads, we still observe many peaks (Fig. 1C).

### 3. Distribution of local signal enrichment

**Fig 2:**
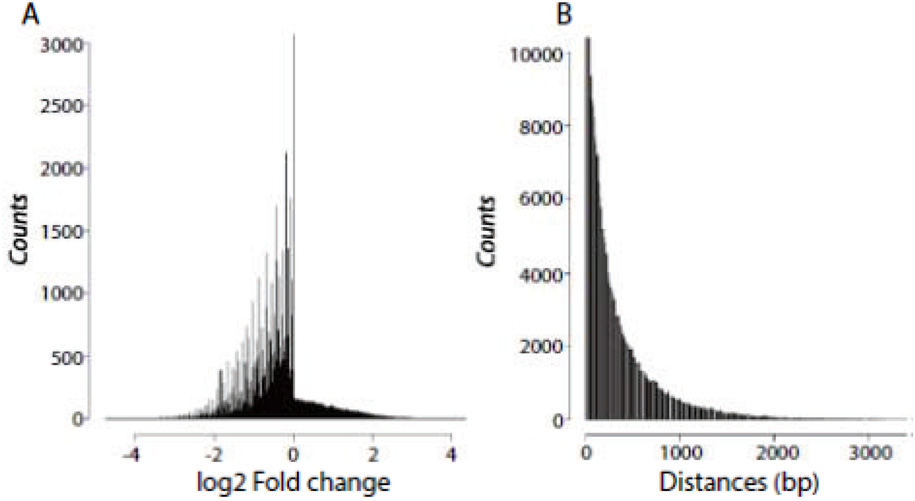
Distributions of local signal enrichment (log2 fold changes) and *Dpnl* cutting sites in the *Drosophila* genome. A: We resampled 90% of the control (Dam only) reads each time, followed by reads scaling and local kernel smoothing at 100-bp running windows for the treatment and control, respectively. We then calculated log2 fold changes in each running window. B: Distribution of the distances in base pairs between adjacent *Dpnl* cutting sites in the *Drosophila melanogaster* genome.

Genome-wide distribution of local signal enrichment is illustrated by a Dam-DsxF sample contrasting with a corresponding Dam only (Fig. 2A). A similar distribution has been observed for Dam-DsxM (data not shown). The distribution reveals an interesting pattern; that is, on the left side the log2 fold changes are markedly variable, which is in sharp contrast to the right side. We suspect that when the DamID signals are weaker than the background signals, the latter vary markedly. However, when the DamID signals are stronger than the background signals, the latter have a small variation across windows.

To test the conjecture in the observation, we then resampled 90% of the control reads each time, followed by reads scaling and computing log2 fold change across the windows. In each iteration of bootstrap resampling, we observed the same distribution as shown in Fig. 2A. This indicates that the feature of background signals is independent of sequence depth, which supports our idea of estimating the distribution of the MFC by bootstrap resampling.

### 4. Identification of significant windows followed by peak calling

After iterative resampling and retrieving the MFC statistic as described in the Method section, we then obtained a distribution of the statistic. Using the 95 percentile as a cutoff, we identified windows with significant (p< 0.05) local signal enrichment. The thresholds are 0.77 and 0.90 for the Dam-DsxF replicates 1 and 2, respectively. Similar statistics (0.73 and 0.79) have been observed for the Dam-DsxM replicates 1 and 2, respectively.

Using the entire quality sort sequence reads that are uniquely mapped in the control, we recomputed the local signal enrichment across the genome-wide windows, in order to identify significant windows. Thresholding the windows, we identified 80,000 ~ 85,000 significant (p < 0.05) windows for each replicate.

In DamID-Seq, distribution of transcription factor binding sites depends on that of *Dpnl* cutting sites in the genome. In order to assess genome-wide distribution of *Dpnl* cutting sites in *Drosophila*, we computed the distances between pairwise adjacent cutting sites.

It is suspected that positions of the induced adenine methylation by the fusion protein follow a Poisson process. Correspondingly, the distances between adjacent *Dpnl* cutting sites exhibit an exponential distribution (Fig. 2B). In fact, as high as 95% of the distances are less than one Kb. These mechanisms determine distributional characteristics of significant windows in the genome; that is, they occur in clusters.

**Fig 3:**
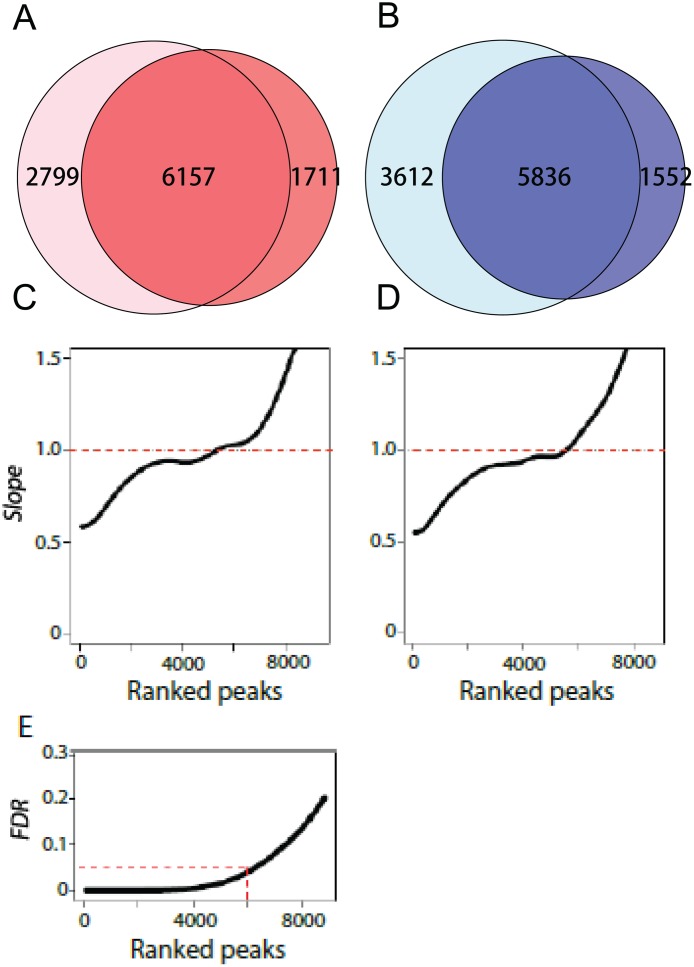
Number of peaks validated by the IDR analysis. A&B: Using the NPPC algorithm, we identified 6,157 and 5,836 between-replicate shared peaks for DsxF and DsxM, respectively. C&D: IDR analysis between biological replicates in DsxF and DsxM, respectively. According to Li et al. [8], a correspondence curve describes the function between a parameter of top t% ranked peaks and corresponding rank intersection between the two replicates. The slope (y-axis) is the first derivative of the function against the parameter. The slope change from 0 to larger than 0 represents the decay point of inconsistency. E: The relationship between ranked peaks and the overall IDR. Corresponding to the top 6,000 peaks, the IDR is 5%.

To call peaks, we then merged the adjacent significant windows by filling the gaps up to one Kb. This will be further justified by comparisons of peaks between DamID-Deq and ChIP-Seq platforms. We identified 6,157 and 5,836 significant (p<0.05) peaks for Dam-DsxF or Dam-DsxM, respectively (Fig. 3 A&B).

### 5. Assessment for DamID-Seq peaks

IDR analysis is a gold standard for peak assessment [14]. As described in the Method section, IDR analysis is based on the pool of both significant and non-significant peaks. We have 10,700–11,500 such total peaks for each replicate per genotype (DsxF and DsxM). In order to compare peaks from different biological replicates of the same genotype, we need two sets of overlapping peaks (≥ 50% bp overlapped). Among the total peaks, overlapping peak pairs in DsxF and DsxM are 8,500 and 8,000, respectively.

IDR analysis is to find the decay point where inconsistency between two replicates occurs [8]. In practice, Li et al. [15] proposed to find the point where slope of the correspondence curve changes from 0 to larger than 0 [8]. In light of this, we have identified 6,000 peaks that are reproducible between the two replicates in DsxF (Fig. 3C). A similar number (5,800) of reproducible peaks has been detected for DsxM (Fig. 3D). Corresponding to the top 5,800–6,000 peaks, the overall IDR is 5% (Fig. 3E). Interestingly, these numbers are congruent to those identified by our algorithm (Fig. 3A&B). Further, these peaks comprise the majority of the physically complete overlapping peaks (Figure A in S1 File).

**Fig 4:**
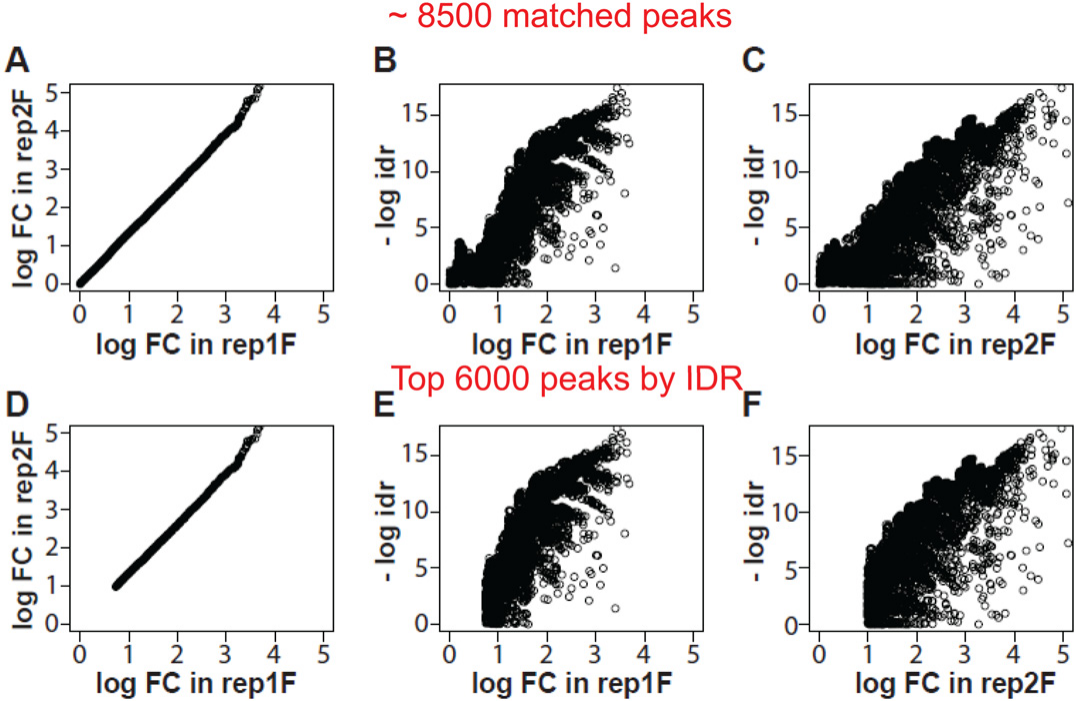
Relationships between local irreproducible discovery rate (idr) and log2 fold changes. Local idr is the probability that two peaks are irreproducible. We use two sets of peaks to illustrate the intrinsic association of local idr and log2 fold changes in DsxF: the set of 8,500 overlapping peaks (≥ 50% bp) between the two biological replicates (A through C), and the set of 6,000 reproducible peaks by IDR analysis (D through F). A and D: Plots of the log2 fold changes between the two replicates. B and E: Plots of the log2 fold changes against the local idr (in -log2 scale) in replicate 1. C and F: Similar plots in replicate 2. Comparisons of the two sets of peaks indicate that IDR analysis is essentially to find the low end cutoff of the log2 fold changes.

We further explored the impact of different peak sets on the relationship between local idr and log2 fold changes. Local idr is the probability that two peaks are irreproducible, which is similar to the concept of local false discovery rate (fdr) [16]. We focused on two sets of peaks in DsxF: the set of 8,500 peaks that are physically overlapping (≥ 50% bp) between the two replicates; and the set of 6,000 peaks that are reproducible. Comparisons of the two peak sets indicate that the relationships of local idr and log2 fold changes differ only at the low end (Fig. 4). This can be translated that IDR analysis is essentially to find the low end cutoff of signal enrichment, or log2 fold changes. This is consistent to the idea that a peak with stronger signal enrichment is more reproducible than a weaker one [8]. Regarding the 6,000 reproducible peaks in DsxF, the low end log2 fold change cutoff is 0.7 and 0.9 for replicates 1 and 2, respectively (Fig. 4 E&F). These values are similar to the thresholds determined by our algorithm. Similar results are obtained for DsxM replicates 1 and 2, respectively (data not shown). These have demonstrated that our peak calling algorithm and IDR analysis are able to cross-validate one another.

### 6. Comparisons of the peaks to those identified by ChIP-Seq

Comparisons of peak sets from different technological platforms can help identify consistent DSX binding sites. Before the DamID-Seq experiments, we conducted ChIP-Seq experiments using *Drosophila* S2 cells [17]. A DSX-specific antibody was used to pull down the DSX protein that is cross-linked to chromatin. A control without the antibody (Input) was used to estimate the background noise. We then applied the SPP algorithm [4] for peak calling and 6,701 and 5,512 peaks were identified for DsxF and DsxM, respectively. These numbers of peaks are similar to those identified by our algorithm in DamID-Seq (Fig. 3A&B). Further, 85% of the ChIP-Seq peaks overlap with the DamID-Seq peaks (> 50% bp). The similarities are illustrated by peak width (Figure B in S1 File) and peak locations (Figure C in S1 File). Similar results have been observed by comparisons to the MACS peak calling algorithm (S3 Fig. and Figure C in S1 File). Taken together, DamID-Seq peaks are comparable to ChIP-Seq peaks.

### 7. Peak distributions relative to gene features

**Fig 5:**
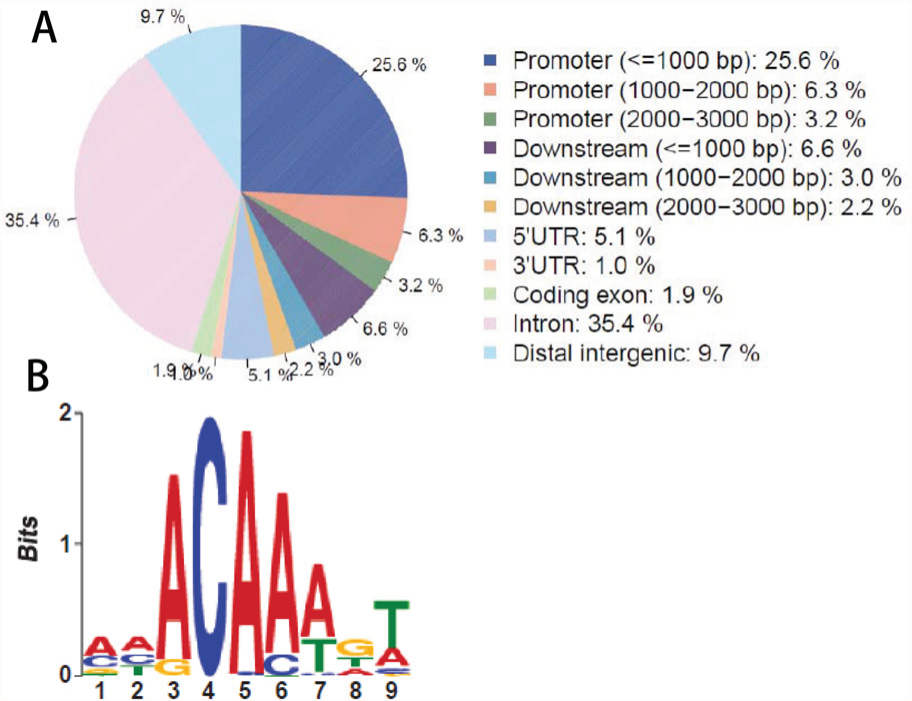
Distribution of peaks in gene features. A: On the basis of the *Drosophila melanogaster* gene annotation (Flybase.r5.38), we aligned the approximately 6,000 peaks of DsxF against the gene features. The majority of the peaks fall into less than one Kb promoters and intron regions, accounting for 61% together; followed by distal intergenic regions (9.7%) and 5′ UTR regions (5.1%). B: In order to identify the sequence motif of DSX binding sites, we performed a *de novo* search using the MEME algorithm (http://meme.nbcr.net/meme/), based on the middle 200-bp DNA sequences of the reproducible peaks.

It is interesting to understand the distribution of the peaks in terms of gene features. On the basis of the *Drosophila melanogaster* gene annotation (Flybase.r 5.38), we mapped the 6,157 Dam-DsxF peaks onto the genome. Results indicate that 35.4% of the peaks falls into intron regions, followed by 25.6% in less than one Kb promoters (Fig. 5A). These two parts together account for 61%. A moderate percentage (9.7%) of the peaks falls into distal intergenic regions (9.7%), followed by 5′ UTR regions (5.1%) (Fig. 5A). Similar distributions have also been observed in Dam-DsxM peaks (data not known). These make sense because DSX is a transcription factor that binds to specific sites in regulatory regions.

### 8. Candidate target genes and binding sequence motif

It is challenging to associate the peaks identified with *Drosophila* genes. We define a gene region as including the gene body and its upstream 2.5 Kb sequence. We then counted number of peaks falling into each gene region. Consider that the majority of the DSX binding sites are shared by both sexes, we then use a criterion of at least one peak observed in each sex or ≥ 2 peaks in one sex. Accordingly, we identified 1,225 genes that are potential DSX target genes (S2 Table). Known DSX target genes, such as yolk protein genes (*yp1* and *yp2*) and *bab1*, have been identified by the NPPC algorithm (Figures D and E in S1 File).

It is interesting to understand functional annotation of the 1,225 candidate target genes. We performed a gene set enrichment analysis using the DAVID Bioinformatics Resources 6.7 developed by NIAID/NIH (http://david.abcc.ncifcrf.gov). Among the top functional clusters include post-embryonic morphogenesis and organ development (Bejamini-Hochberg [18] adjusted p < 3.4X10^-26^), tissue morphogenesis (p < 7.8x10^-21^), and imaginal disc morphogenesis (p < 5.9x10^-18^). These are significant functional annotations related to the differences in fat body between female and male *Drosophila*, which make sense because DSX plays an important role in sex determination.

In order to identify the sequence motif of DSX binding sites, we performed a *de novo* search using the MEME algorithm (http://meme.nbcr.net/meme/cgi-bin/meme.cgi). We extracted the DNA sequences from the Flybase (http://flybase.org/), based on the coordinates of the reproducible peaks. A sequencing motif has been identified (Fig. 5B), which is similar to a previous report [9].

## Discussion

We demonstrated that DamID-Seq is a technological platform that is comparable to ChIP-Seq for genome-wide identification of transcription factor binding sites. ChIP-Seq is a commonly used technology that depends on availability of highly specific antibodies. DamID-Seq is a new technology that seems to be promising. But background signals need to be carefully controlled using statistical methods as shown in this study. Given this, we are able to identify DamID-Seq peaks similar to those by ChIP-Seq in terms of peak numbers, width, and positions.

Previous peaking calling algorithms, including SPP and MACS that were designed for symmetrical signal distributions, are powerful for ChIP-Seq data [3,4]. The symmetry is assured by the DNA double-strand structure. In DamID-Seq experiments, however, induced adenine methylation at GATC sites are not guaranteed to be symmetrical, because the chemical process is affected by many known and unknown factors, for instance, 3-D structure of the chromatin (S4 Fig.). We observed that when calling peaks using SPP and MACS, well established DSX target genes, including *yp1* and *yp2*, are inconsistently tagged by a peak between replicates (S2 Fig.). This suggests that the methylation status might even differ between different biological replicates of the same genotype. However, these known target genes are consistently called and tagged by the NPPC algorithm.

We have proposed a statistical method for controlling the background signals in DamID-Seq data. This method is based on reads resampling, in order to estimate the averaged behavior of the background signals. Results from our algorithm (NPPC) have been cross-validated by IDR analysis, which has been suggested as a gold standard by the EDCODE consortium [14]. Although IDR analysis and the NPPC algorithm differ in the starting points of parametric vs. nonparametric considerations, they are complementary to one another by forming a reciprocal validation cycle. These indicate that the NPPC algorithm is robust in controlling the signals of background adenine methylation in DamID-Seq. The NPPC algorithm is coded in R (www.cran.r-project.org) and freely available upon request.

The NPPC algorithm is based on resampling reads in the control. An alternative way is to resample reads in the treatment, if the background signals are uniformly distributed. The random distribution of mapped reads in the control convinced us that the background noises need to be accurately estimated. This method may be adapted to other technologies, such as RIP-Seq, which rely on comparisons to a control with a similar background noise distribution.

It is suspected that positions of the induced adenine methylation by the fusion protein follow a Poisson process. Correspondingly, the distances between adjacent *Dpnl* cutting sites follow an exponential distribution. In fact, as high as 95% of the distances are less than one Kb. These mechanisms determine distributional characteristics of significant windows in the genome; that is, they occur in clusters. We then proposed to merge the adjacent windows with significant local signal enrichment, filling the gaps up to one Kb. This is justified by the similarities of peaks called by different algorithms, based on data from different technological platforms.

## Supporting Information

S1 Fig A flow chart of the NPPC peak calling algorithm. (PDF)

S2 Fig KNown DSX target genes are inconsistently called by SPP between replicates. A: Coordinates around the yp1 and yp2 gene regions. B and C: Signal (background subtracted) distributions of the yp1 and yp2 gene regions between two respective replicates of the same DsxF genotype. D. A peak (represented by a horenzontal black bar) is called for replicate 1, but not called by replicate 2. E. Gene symbols and location in the region.
(PDF)

S3 Fig Comparisons of peak width between DamID-Seq and ChIP-Seq. We used the Dsx-specific antibody to perform the ChIP-Seq experiments based on the S2 cell lines. On the basis of the data, we then call 7,201 and 6,021 peaks for DsxF and DsxM, respectively, using the MACS algorithms [4]. These peaks are compared to the DamID-Seq peaks called by the NPPC algorithm. In general, the median peak width is larger by the the MACS algorithm on Chip-seq, compared to the peaks called by the NPPC algorithm on DamID-Seq. The variations of peak width are similar.
(PDF)

S4 Fig A figure illustrating that the 3-D chromatin structure may impact the location of adenene methylation at GATC sites. (PDF)

S1 File Figure A, Overlapping ratio of peaks. Overlapping rate is the percentage of overlapped base pairs relative to the smaller peak in a pair for comparison. A rate of 0 means no overlapping and a rate of 1 means completely overlapping. A and B are overlapping rates between two replicates in Dam-DsxF and Dam-DsxM, respectively. Approximately 5,000 peaks exhibit complete overlap in each of the two genotypes. Figure B, Comparisons of peak width between DamID-Seq and ChIP-Seq. We used the Dsx-specific antibody to perform the ChIP-Seq experiments based on the S2 cell lines. On the basis of the data, we then call 6,701 and 5,512 peaks for DsxF and DsxM, respectively, using the SPP algorithms [4]. These peaks are compared to the DamID-Seq peaks called by the NPPC algorithm. In general, the median peak sizes are similar, but the variation of peak sizes in DamID-Seq is larger than that in the ChIP-Seq. Figure C, Consistent peak locations detected by both DamID-Seq and ChIP-Seq. This is an example to illustrate the consistent peak location at the promoter region of *Hsc70* gene. Figure D, A known DSX target gene ***bab1*** is identified by the NPPC algorithm. Figure E, Known DSX target genes ***Yp1*** and ***Yp2*** are identified by the NPPC algorithm.
(PDF)

S1 Table Sequencing depth summary. (DOC)

S2 Table Potential DSX target genes. (XLS)
