# Supplementary figures and images for: A Non-Parametric Peak Calling Algorithm for DamID-Seq
Source: PLoS One. 2015 Mar 18;10(3):e0117415. doi: 10.1371/journal.pone.0117415 (PMC4364623; doi:10.1371/journal.pone.0117415)

# Supplemental Figure 1

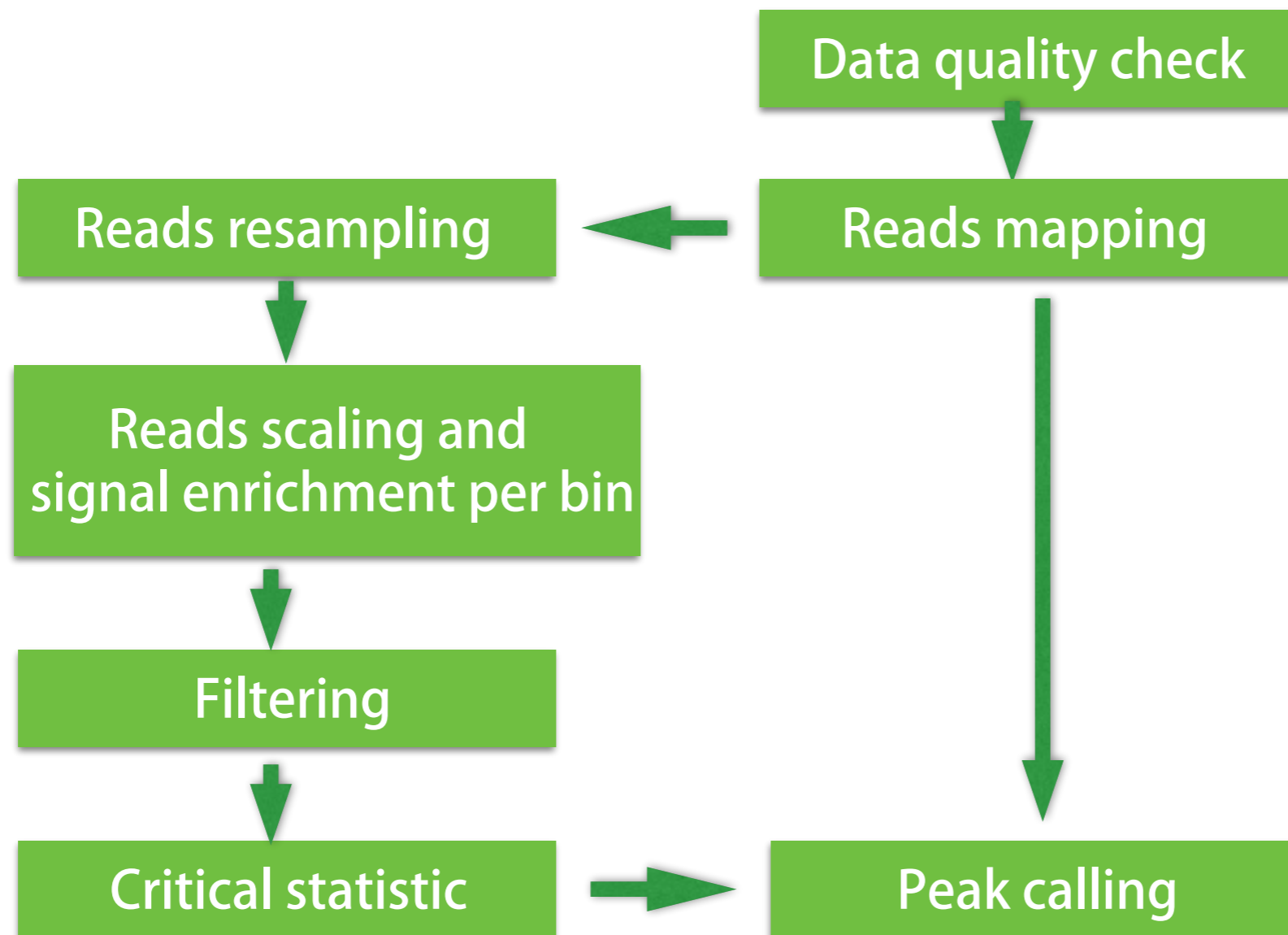

Supplement: S1 Fig — (PDF) [file pone.0117415.s001.pdf]

Supplemental Figure 2

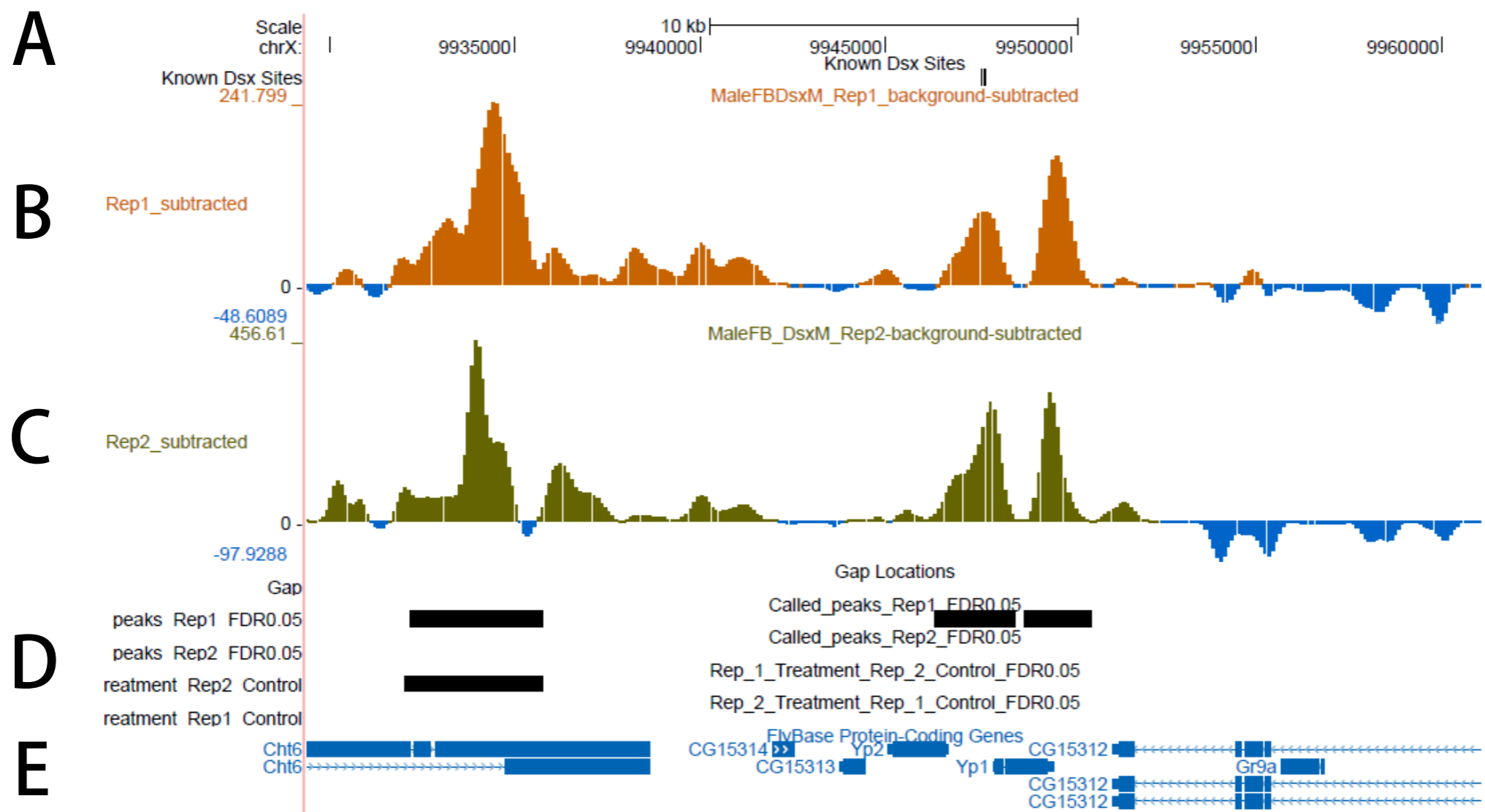

Supplement: S2 Fig — A: Coordinates around the yp1 and yp2 gene regions. B and C: Signal (background subtracted) distributions of the yp1 and yp2 gene regions between two respective replicates of the same DsxF genotype. D. A peak (represented by a horenzontal black bar) is called for replicate 1, but not called by replicate 2. E. Gene symbols and location in the region. (PDF) [file pone.0117415.s002.pdf]

Supplemental Figure 3

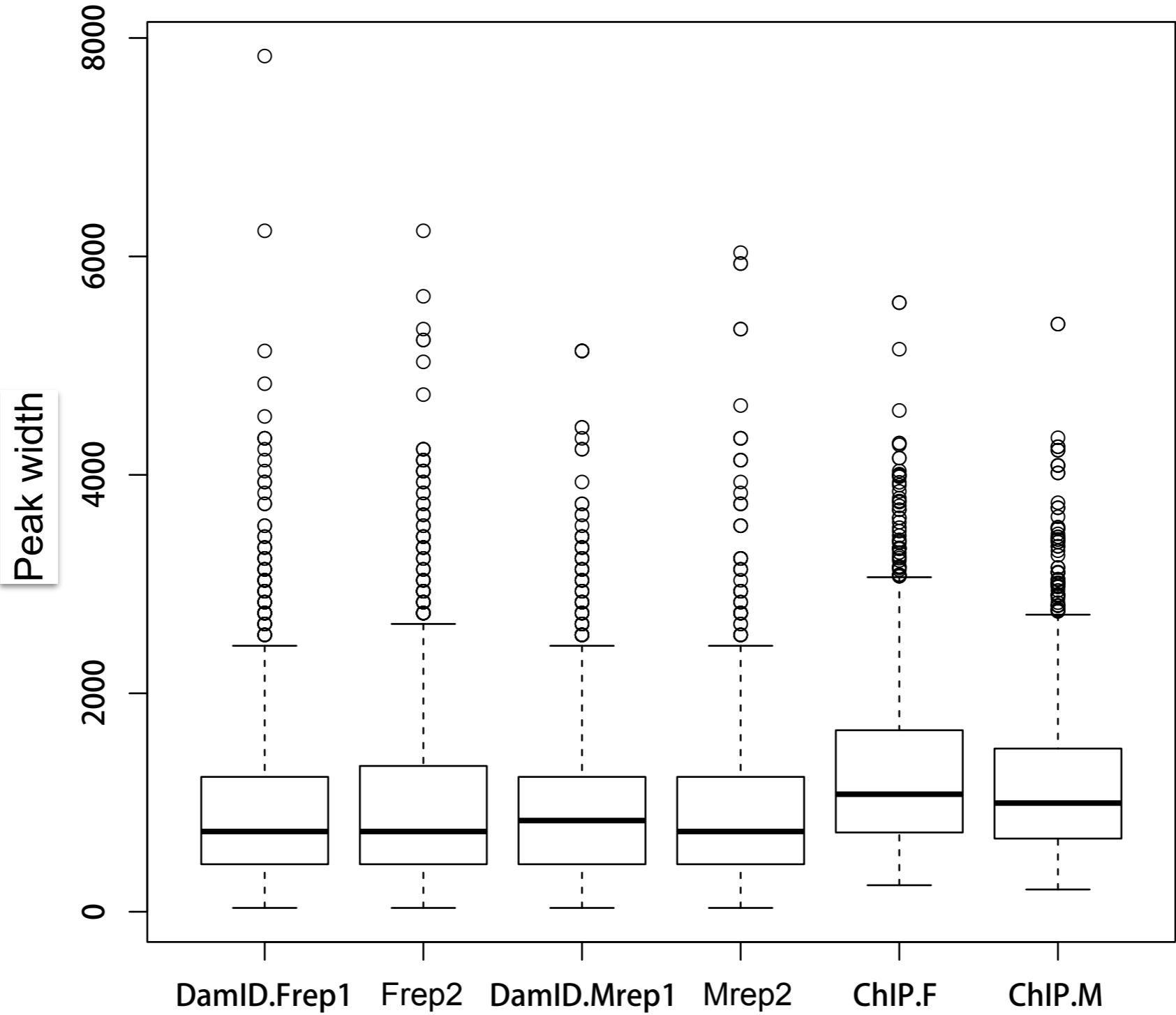

Supplement: S3 Fig — We used the Dsx-specific antibody to perform the ChIP-Seq experiments based on the S2 cell lines. On the basis of the data, we then call 7,201 and 6,021 peaks for DsxF and DsxM, respectively, using the MACS algorithms [4]. These peaks are compared to the DamID-Seq peaks called by the NPPC algorithm. In general, the median peak width is larger by the the MACS algorithm on Chip-seq, compared to the peaks called by the NPPC algorithm on DamID-Seq. The variations of peak width are similar. (PDF) [file pone.0117415.s003.pdf]

Supplemental Figure 4

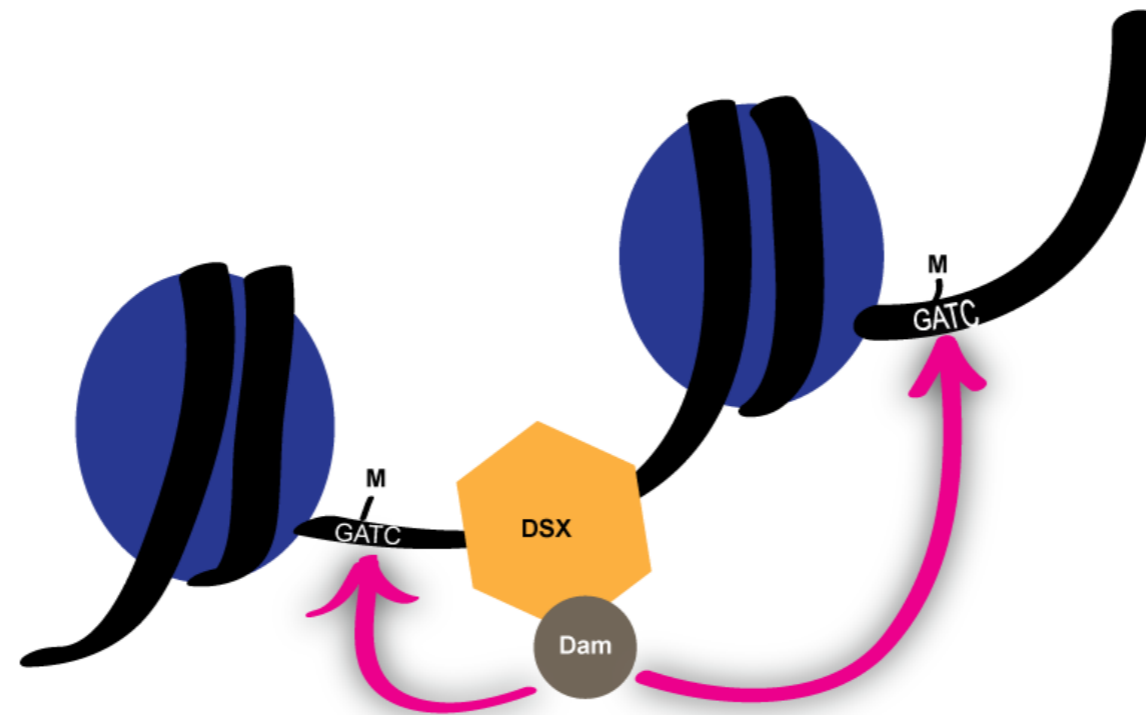

Supplement: S4 Fig — (PDF) [file pone.0117415.s004.pdf]
