# Supplementary material for: A Non-Parametric Peak Calling Algorithm for DamID-Seq
Source: PLoS One. 2015 Mar 18;10(3):e0117415. doi: 10.1371/journal.pone.0117415 (PMC4364623; doi:10.1371/journal.pone.0117415)
Supplement: S1 File — A rate of 0 means no overlapping and a rate of 1 means completely overlapping. A and B are overlapping rates between two replicates in Dam-DsxF and Dam-DsxM, respectively. Approximately 5,000 peaks exhibit complete overlap in each of the two genotypes. Figure B, Comparisons of peak width between DamID-Seq and ChIP-Seq. We used the Dsx-specific antibody to perform the ChIP-Seq experiments based on the S2 cell lines. On the basis of the data, we then call 6,701 and 5,512 peaks for DsxF and DsxM, respectively, using the SPP algorithms [4]. These peaks are compared to the DamID-Seq peaks called by the NPPC algorithm. In general, the median peak sizes are similar, but the variation of peak sizes in DamID-Seq is larger than that in the ChIP-Seq. Figure C, Consistent peak locations detected by both DamID-Seq and ChIP-Seq. This is an example to illustrate the consistent peak location at the promoter region of Hsc70 gene. Figure D, A known DSX target gene bab1 is identified by the NPPC algorithm. Figure E, Known DSX target genes Yp1 and Yp2 are identified by the NPPC algorithm. (PDF) [file pone.0117415.s005.pdf]

Supplemental Figure A

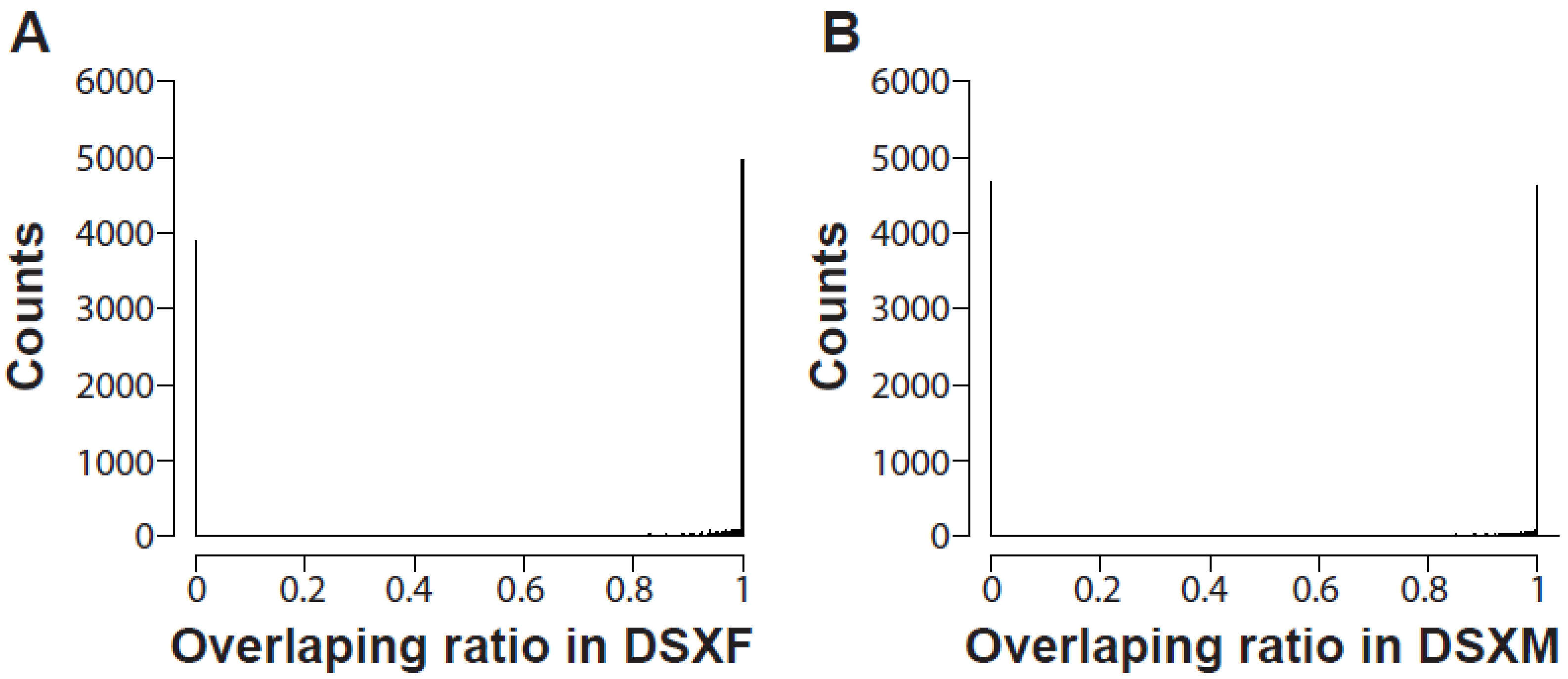

Supplemental Figure B

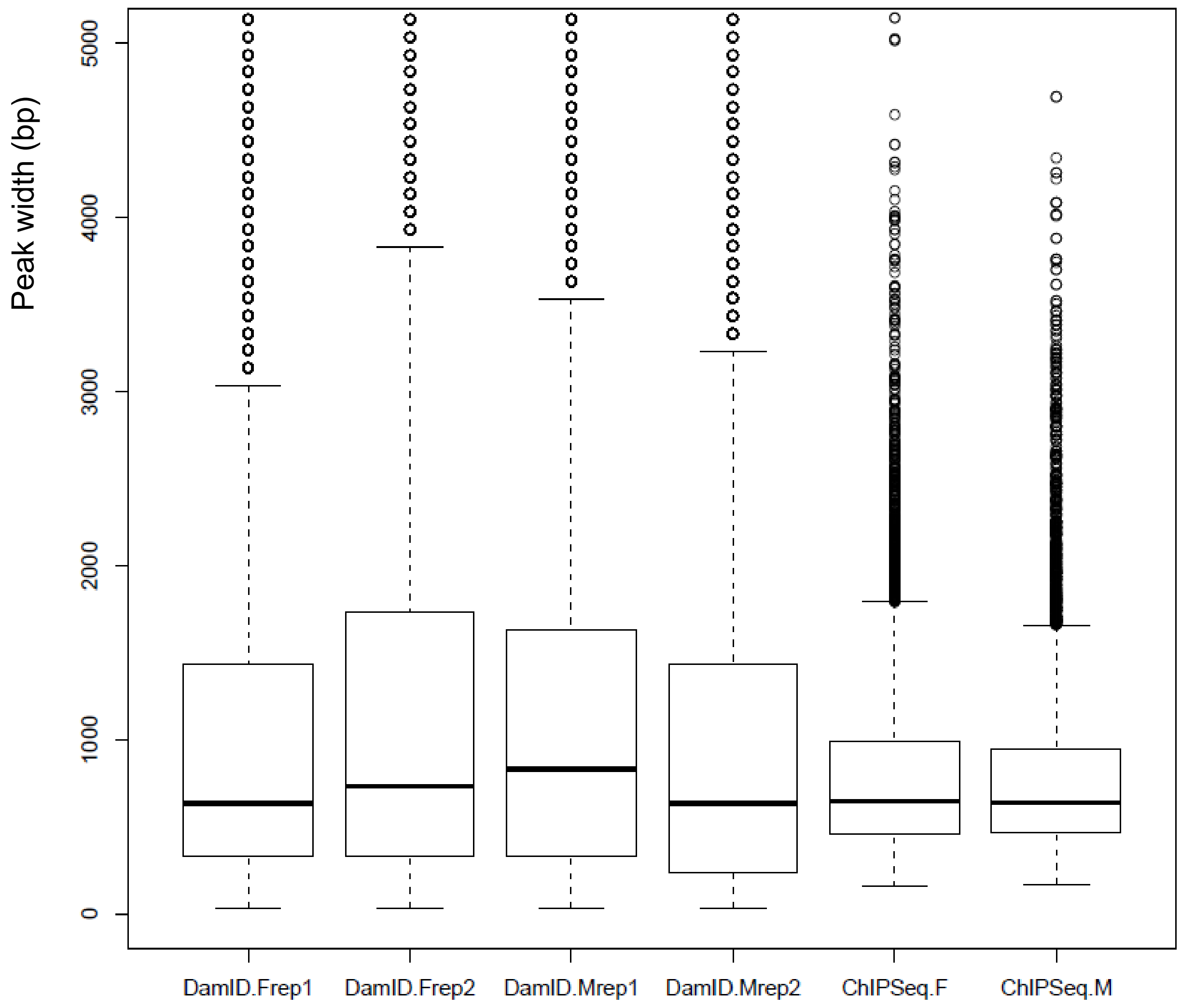

Supplemental Figure C. Consistent peak locations tagging Hsc70 g

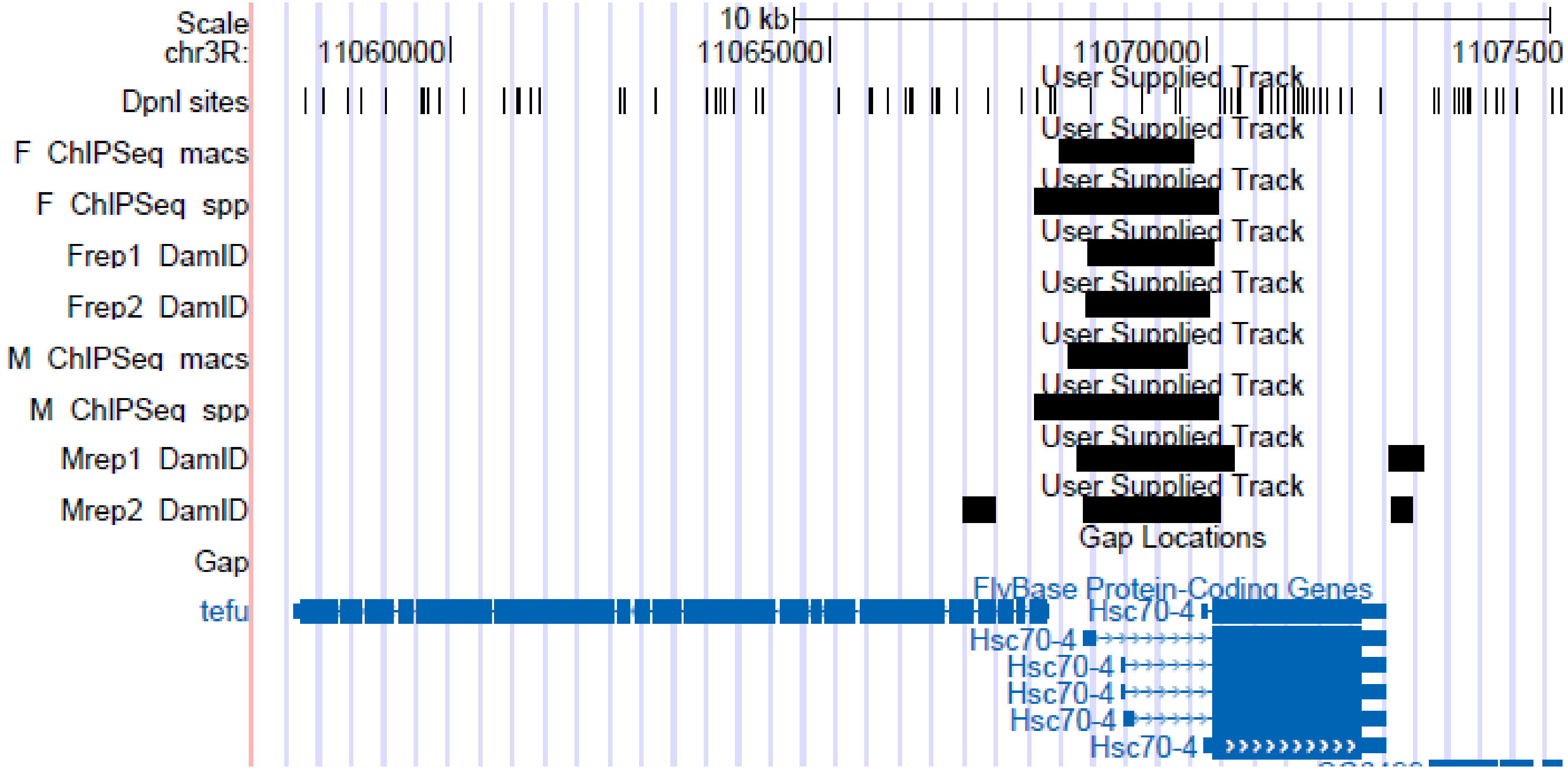

Supplemental Figure D

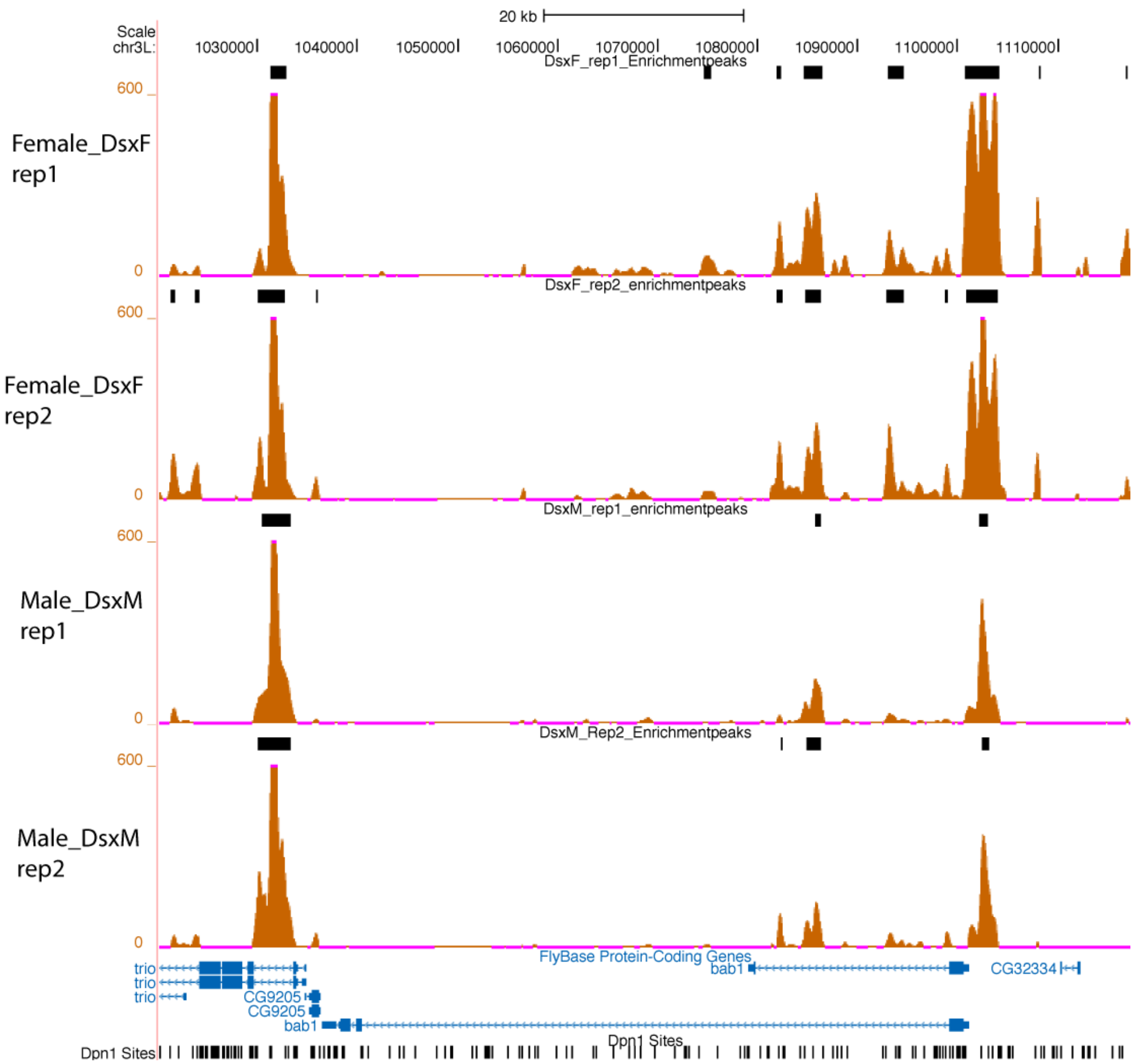

Supplemental Figure E

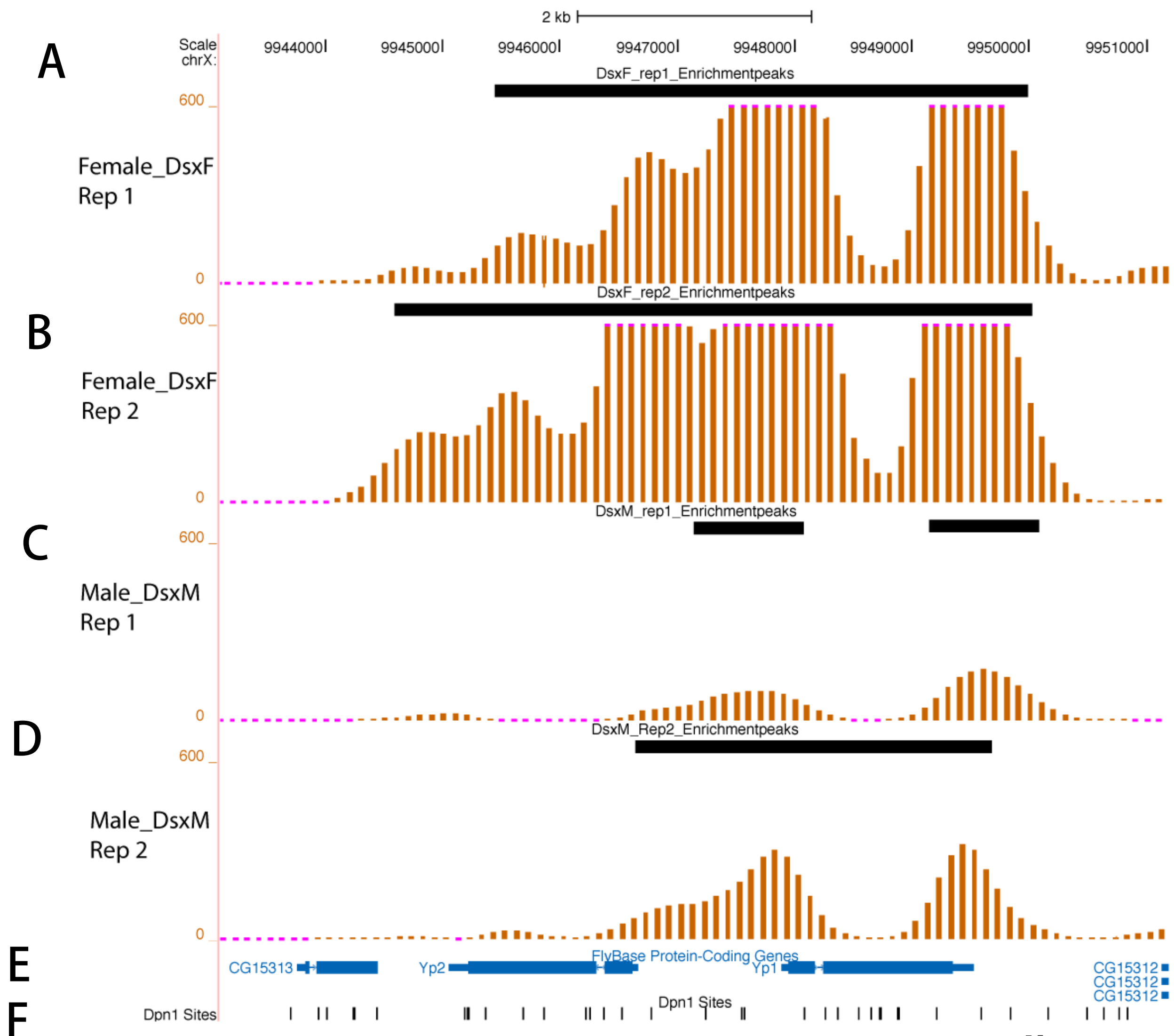

Yolk Protein Genes
