# Supplementary material for: A Non-Parametric Peak Calling Algorithm for DamID-Seq
Source: PLoS One. 2015 Mar 18;10(3):e0117415. doi: 10.1371/journal.pone.0117415 (PMC4364623; doi:10.1371/journal.pone.0117415)
Supplement: S1 Table — (DOC) [file pone.0117415.s006.doc]

**Supplemental table 1. Sequencing depth summary**

|  | **Total # Reads**  **Trimmed 17 bp** | **# Uniquely aligned reads** |
| --- | --- | --- |
| Male DsxM_1 | 20,013,880 | 16,103,199 (80.5%) |
| Male DsxM_2 | 31,472,062 | 26,627,238 (84.6%) |
| Male Dam_1 | 19,158,137 | 12,773,533 (66.7%) |
| Male Dam_2 | 33,324,254 | 22,304,132 (66.9%) |
| Female DsxF_1 | 34,089,738 | 28,935,213 (84.9%) |
| Female DsxF_2 | 31,509,090 | 27,169,473 (86.2%) |
| Female Dam_1 | 32,940,343 | 22,017,947 (66.8%) |
| Female Dam_2 | 33,638,531 | 21,516,050 (64%) |
